# Human placental perfusion measured using dynamic contrast enhancement MRI

**DOI:** 10.1371/journal.pone.0256769

**Published:** 2021-09-02

**Authors:** Benjamin Deloison, Chloé Arthuis, Gabriel Benchimol, Daniel Balvay, Laurence Bussieres, Anne-Elodie Millischer, David Grévent, Cécile Butor, Gihad Chalouhi, Houman Mahallati, Olivier Hélénon, Bertrand Tavitian, Olivier Clement, Yves Ville, Nathalie Siauve, Laurent Julien Salomon

**Affiliations:** 1 Service de Gynécologie-Obstétrique, Hôpital Necker-Enfants Malades, Assistance Publique-Hôpitaux de Paris (AP-HP), Paris, France; 2 EA fetus 7328 and LUMIERE platform, Université Paris Descartes, Paris, France; 3 INSERM, U970, Paris Cardiovascular Research Center–PARCC, Sorbonne Paris Cité, Paris, France; 4 Service de Gynécologie-Obstétrique, Hôpital mère-enfant, CHU Nantes, Nantes, France; 5 Service de Radiologie, Hôpital Necker Enfants Malades, Assistance Publique-Hôpitaux de Paris (APHP), Paris, France; 6 Service de Radiologie, Hôpital Louis Mourier, Assistance Publique-Hôpitaux de Paris (APHP), Colombes, France; Clinic Hospital of Zaragoza, SPAIN

## Abstract

**Objectives:**

To evaluate the feasibility of dynamic contrast enhanced magnetic resonance imaging (DCE MRI) and measure values of in vivo placental perfusion in women.

**Methods:**

This study was part of the Placentimage trial (NCT01092949). Gadolinium-chelate (Gd) enhanced dynamic MRI was performed two days before termination of pregnancies at 16 to 34 weeks gestational age (GA). Quantitative analysis was performed using one-compartment intravascular modeling. DCE perfusion parameters were analyzed across GA and were compared in IUGR and AGA fetuses.

**Results:**

134 patients were enrolled. After quality control check, 62 DCE MRI were analyzed including 48 and 14 pregnancies with normal and abnormal karyotypes, respectively. Mean placental blood flow was 129±61 mL/min/100ml in cases with normal karyotypes. Fetuses affected by IUGR (n = 13) showed significantly lower total placental blood flow values than AGA fetuses (n = 35) (F _total_ = 122±88 mL/min versus 259±34 mL/min, p = 0.002). DCE perfusion parameters showed a linear correlation with GA.

**Conclusions:**

Measuring placental perfusion in vivo is possible using DCE MRI. Although this study has many limitations it gives us the first DCE MRI values that provide a potential standard for future research into placental perfusion methods and suggests that placental functional parameters are altered in IUGR pregnancies.

## Introduction

The placenta constitutes a circulatory interface between the mother and the fetus, supplying oxygen and nutrients to the fetus. Appropriate development of the placenta is required for the normal growth of the fetus. Placental insufficiency leads to several adverse outcomes in pregnancy, such as intrauterine growth restriction (IUGR) and pre-eclampsia. Pathophysiology of placental insufficiency remains unclear but a common assumption is that placental perfusion decreases early in pregnancy [1, 2].

Ultrasonography is currently used to evaluate fetal growth and morphology during pregnancy. Doppler ultrasonography can evaluate fetal well-being through measurements of various circulatory parameters such as is done in spectral analysis of the flow in the umbilical artery, middle cerebral artery, and ductus venosus. Ultrasonography can also describe the localization and morphology of the placenta, but cannot evaluate its flow and function, which should ideally be measured in ml/min/100mL of placenta. By specifying that the density of the placenta being globally of 1, one can approximate the volume by the placental weight [3]. At most, increased resistance of the uterine artery, measured by Doppler ultrasonography, may indicate a higher risk of placental insufficiency as early as the end of the first trimester of pregnancy [4].

Magnetic Resonance Imaging (MRI) is safe during pregnancy and increasingly used [5]. Functional MRI has already been used to evaluate cerebral, cardiac, tumor and hepatic perfusion [6–8], and could provide new insights into placental function. Our team has previously developed animal models of dynamic contrast enhanced (DCE) MRI [9–14]. The feasibility of DCE MRI has also been demonstrated in primates [15]. Using a one compartment model, placental perfusion and more specifically maternal perfusion of the placenta (F) was successfully measured in ml/min/100mL. DCE MRI could therefore permit the detection of placental insufficiency if due to decreased perfusion in pregnant women before the clinical onset of IUGR or preeclampsia. However, at present DCE MRI is not used in ongoing pregnancies as the safety of Gadolinium (Gd) based contrast agents remains controversial [15–17] in human embryogenesis and contrast enhanced MRI in pregnancy is currently limited to rare clinical scenarios where the maternal benefits far outweigh the unknown fetal risks [18].

The objective of the Placentimage trial (ClinicalTrials.gov, Identifier: NCT01092949) was to preliminary explore the potential of DCE MRI using Gd based contrast in pregnant women as a technique to measure human placental perfusion, and to report the observed ranges of in vivo placental perfusion in our study population. It was based on imaging studies of patients undergoing termination of pregnancy (TOP) at 16 to 34 weeks gestational age GA for fetal indications in a tertiary referral center.

## Methods

### Study design and population

This study was part of the Placentimage trial (ClinicalTrials.gov, Identifier: NCT01092949). Participants reviewed an information sheet and signed an informed consent form. Four French centers were involved in the study between 2010 and 2015, and DCE MRI using Gd was performed prior to TOP to assess placental perfusion in pregnant women. The protocol was approved by the ASNM (Agence Nationale de Sécurité du Médicament) and the CPP (Comité de Protection des Personnes).

All patients undergoing TOP for fetal indications from 16 to 34 weeks GA (gestational age based on 1^st^ trimester measurement of crown–rump length were asked if they wished to participate in this study. Participation in the study, did not change protocol for TOP and MRI findings did not affect patient management. All TOP were performed in accordance with local laws and protocols.

Inclusion criteria: pregnant women older than 18 who were to undergo TOP for fetal indications and who consented to participate in the study.

Exclusion criteria: placental adhesion anomalies suspected on pre-natal ultrasonography, allergy to Gd contrast, any contraindication to MRI, renal insufficiency.

Inclusion of 15 patients per two-week gestational age window, between 16 and 34 GA, for a total of 135 was anticipated. MRI was performed two days before TOP. All MRI studies were anonymized after the imaging was completed.

### Contrast agent

A conventional Gd based agent (Dotarem®, Guerbet, Aulnay-sous-Bois, France) was used at a dose of 0.05 mmol Gd/kg of body weight, i.e. half the clinical dose. An automated injector was used to deliver a constant flow rate of 4 ml/s. Immediately following this, a 20 mL saline flush was administrated at the same rate.

### Imaging protocol

The acquisitions were carried out by experienced technologists. MRI was performed on 1.5 Tesla units with an abdominal coil and a dorsal position used as first choice. Sequences performed in order were balanced steady state gradient echo sequence (FIESTA/TRU-FISP/balanced-FFE on different platforms) in the three conventional orthogonal planes followed by 3D Fast Spoiled Gradient Echo (FSPGR) for the DCE MRI portion of the study. While the MR parameters and devices were slightly different between centers (detailed in the S1 File), these sequences are standard sequences available on all routine clinical MR systems.

### Data analysis

#### Signal to Noise Ratio (SNR) and Contrast to Noise Ratio (CNR)

SNR and CNR were compared qualitatively between the four different centers. SNR is the ratio of true signal from tissue(s) of interest relative to background noise from random signals, whereas CNR describes the difference in signal between two areas relative to the background noise. One can think of CNR of two tissues, a and b, as CNR = SNRa-SNRb.

#### Kinetic SI curves

Kinetic signal intensity curves demonstrate the relative signal intensity in a region of interest plotted over time. MR images were analyzed on a workstation using software (PhysioD3D) [19] that was locally developed using MATLAB (R2012b; MathWorks, USA) [9–14]. Manual segmentation of the arterial input function (AIF) and the placenta was performed. Detail of the segmentation method is reported in the S1 File.

Signal Intensities (SIs) were measured by regions of interests (ROIs) over the aorta (or iliac artery, only when the aorta was not visible on the selected axial slice) and the entire placenta. For simplification, we assumed a linear relationship between contrast enhancement and Gd concentrations. Mean baseline SI before the contrast agent injection (S0) was subtracted from the post-injection SI to obtain kinetic enhancement curves for each ROI [20, 21].

#### Quality criteria of the AIF and the placental curves

Quality criteria of the AIF and the placental curves were established based on physiological assumptions and previous reports (S1 File), and were analysed by blinded radiologists. Given that AIF reflects the concentration of contrast agent over time, the AIF curve of the arterial vessels had to demonstrate an initial peak reflecting the rapid injection of the contrast bolus, then a rapid but partial decrease, and followed by a more tapered decrease. Given that placental enhancement curves reflect the transit of contrast through the vascular bed and tissue, they had to be more gradual and progressive, without an acute peak, and with a steady state below the AIF curve at the end of the acquisition (S1A Fig)

#### Compartmental analysis

**Fig 1:**
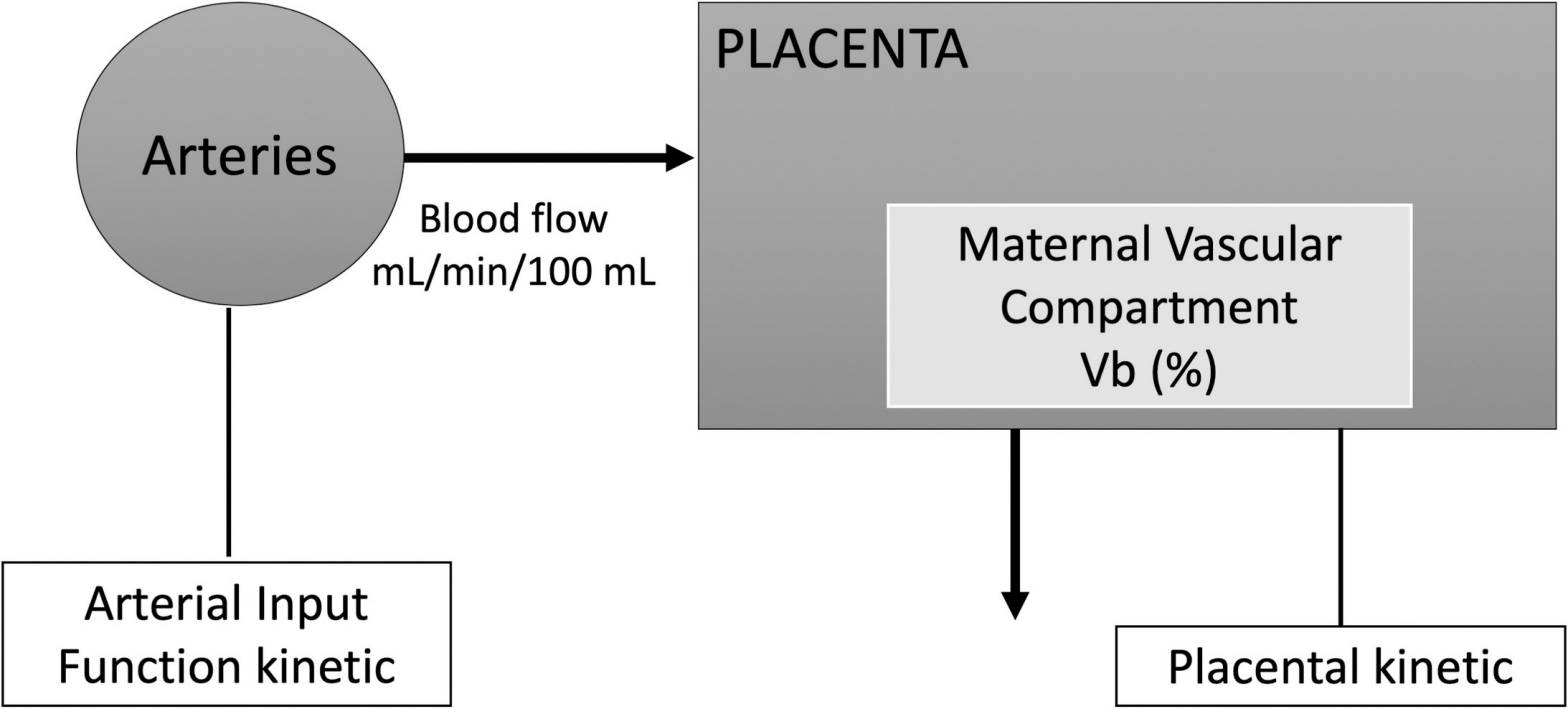
One-compartment model.

We used a one-compartment model based on physiological assumptions and previous reports (Fig 1) [9–13, 21–23] which assumes that the placenta is supplied by an arterial input (uterine arteries) and drained solely by venous output, and that the compartment volumes remain stable during the imaging experiment. Gd leakage into the amniotic fluid and fetus was neglected, as it was not measurable during the experiment.

The variation of the amount of contrast agent in the placenta was described by the following equation [23]:

$$\frac{dq_{placenta}}{dt}=F.q_{aorta}(t-Dt)-\frac{F}{V_{b}}.q_{placenta}$$ (1)

where q_placenta_ was the amount of Gd in the blood compartment per unit of time, q_aorta_ was the amount of Gd in the maternal aorta per unit of time (or iliac artery, only when the aorta was not visible on the selected axial slice), and Dt (seconds) is the time between the start of arterial enhancement and start of tissue enhancement. Four perfusion parameters were calculated: F, placental blood flow per unit tissue volume (in mL/min/100mL), F_total_, placental blood flow for the whole placenta (in mL/min) V_b_, blood volume fraction of the maternal vascular placental compartment (in %) and MTT (Mean Transit Time), average time that the Gd spends in the placenta (in seconds). F_total_ was calculated by multiplying F by the placental weight (in grams) reported at delivery. V_b_ was the ratio between placental blood volume and placental volume. MTT was the ratio between V_b_ and F. Outliers (values below or above -4SD or 4SD) were excluded.

### Statistical analysis

Results were interpreted according to karyotype results and growth (IUGR and appropriate for gestational age birthweight AGA). Birthweight (g) and placental weight (g) were recorded at delivery. Birth weight was transformed into a Z_score_ for GA [24]. At birth weight Z_score_ < -1.28 was considered to reflect possible IUGR. Population characteristics were compared by ANOVA.

Relationships between DCE (F, F_total_, V_b_ and MTT) and fetal parameters (GA and birth weight) were investigated. Linear regression between placental perfusion parameters and GA or birth weight were performed first assessed in x–y plots and then by best curve fit. Given the confounding effects, fetuses with chromosomal anomalies were excluded from the placental perfusion analysis and/or IUGR were not included in determining the ranges for placental perfusion (as placental insufficiency and decreased perfusion were more likely in these settings) nor the relationship between placental function and GA. The student t-test was used to compare perfusion parameters between fetuses with IUGR and non-IUGR AGA fetuses. Results of the pathological exams of placentas were analysed in each groups. Significant placental abnormalities were defined as the presence of a combination of intervillous thrombosis, infarcts, villous necrosis, fibrin deposition, thrombosis, hematoma or massive subchorial thrombosis [25]. All tests were two-tailed and p-values <0.05 were considered statistically significant.

## Results

### Patients

**Fig 2:**
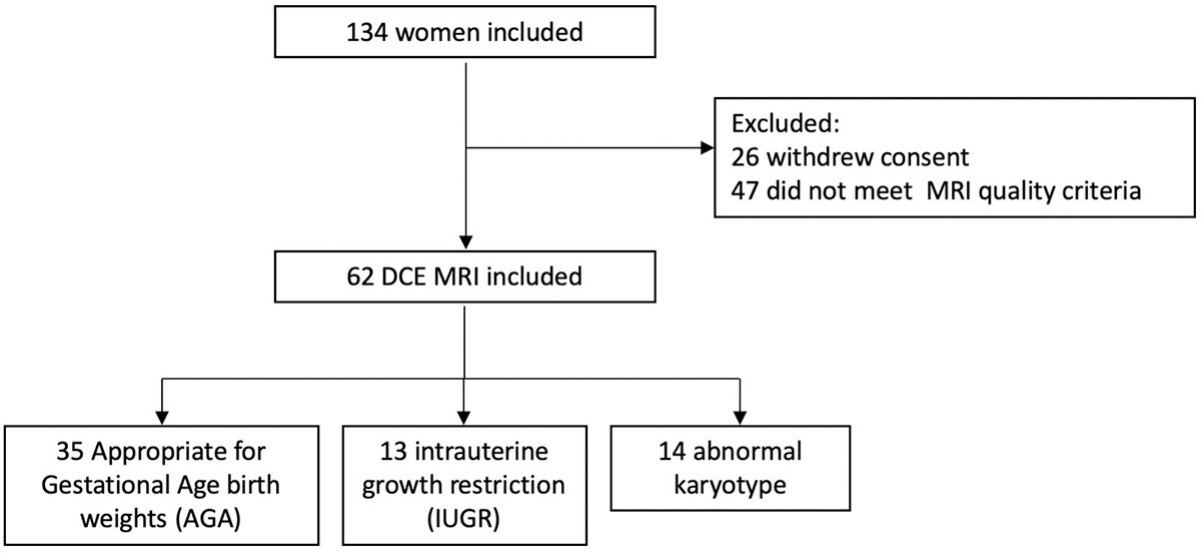
Flowchart.

134 patients were initially enrolled in the study (Fig 2). The main reasons for TOP were central nervous system anomalies 35 (26.1%), chromosomal anomalies 28 (20.9%), congenital heart defects 22 (16.4%), skeletal malformations 12 (8.9%), genito-urinary malformations 11 (8.2%), and polymalformations 10 (7.4%). No cases of preeclampsia were found among the patients included in the study.

26 patients withdrew their consent prior to the MRI examination. Therefore, 61, 40, 5 and 2 MRI studies were performed in the four centers. Other than nausea, vomiting, and nonspecific patient discomfort, no adverse outcome was reported during the trial.

### Analysis of the CNR and SNR

**Fig 3:**
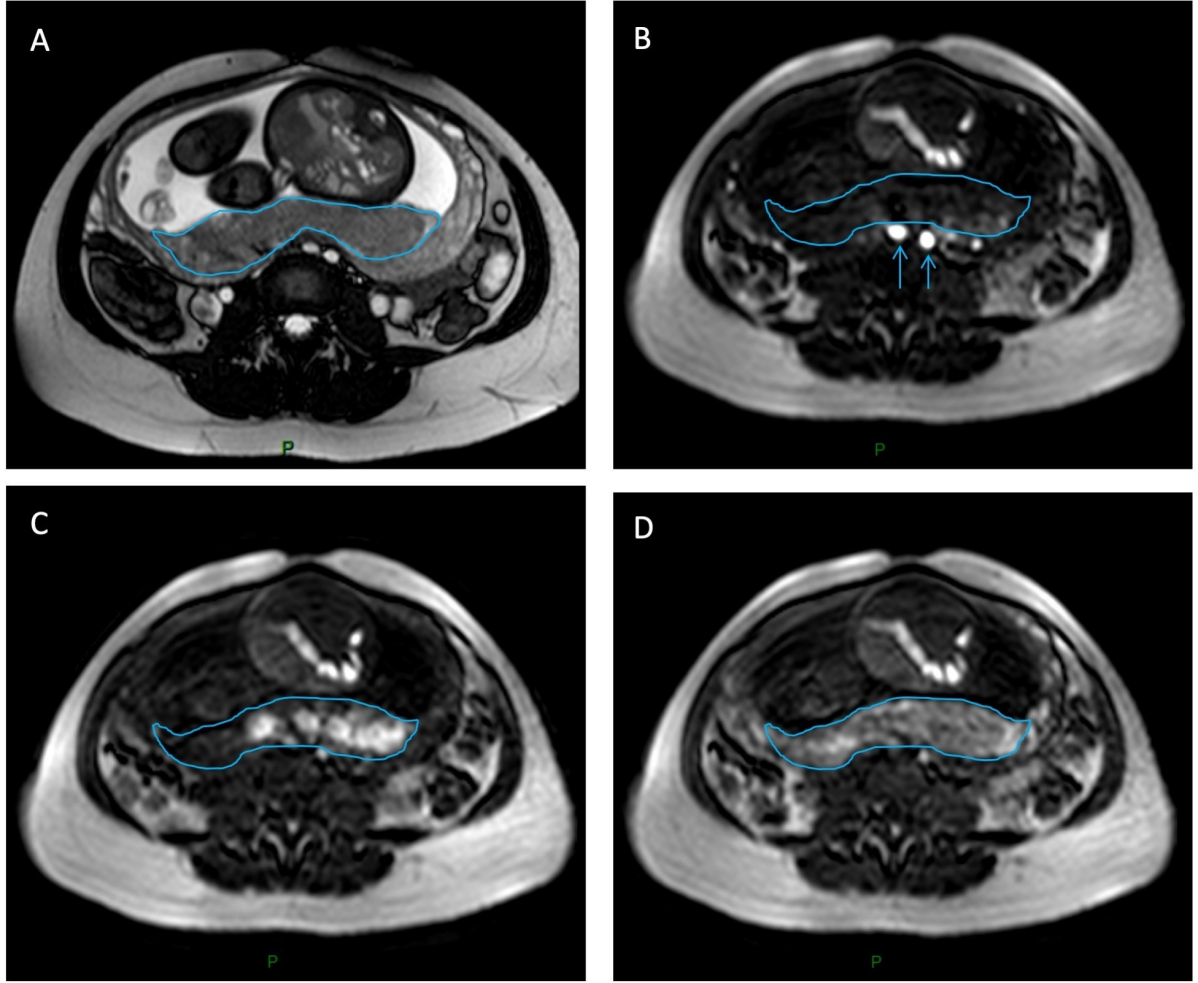
DCE MRI in the axial plane. **A**, shows a morphologic SSFP/FIESTA/TRU-FISP sequence. **B**, FSPGR image showing the enhancement in the iliac arteries after an intravenous bolus Gadolinium-chelate (blue arrows) and **C, D**, the enhancement of the placenta appears as high signal on these T1 FSPGR images (blue outline). After compartmental analysis of the enhancement, functional DCE MRI parameters could be evaluated.

Fig 3B–3D shows a set of FSPGR images for perfusion imaging.

A qualitative review of CNR and SNR demonstrated center 1 to have the highest SNR and CNR of all 4 centers. While large voxel sizes at center 3 led to poor image resolution and significant pixelation of images, subjectively the SNR and CNR were only slightly inferior at this center. Center 2 and 4 both had the lowest SNR.

### Analysis of the kinetic curves

**Fig 4:**
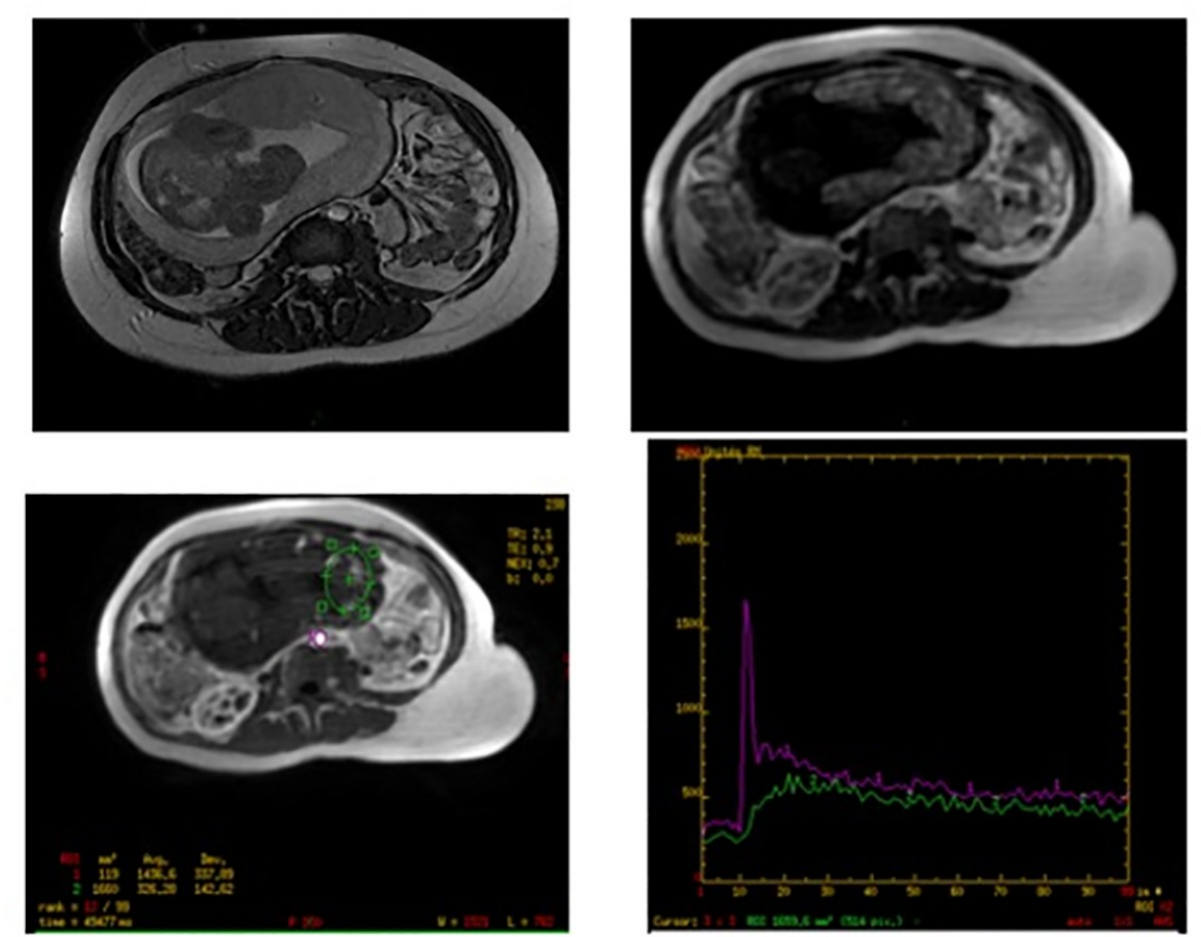
Example DCE time activity curve with model fit from a relevant placental region of interest.

In 63 cases (61 from center 1; 2 from center 4), kinetic curves of AIF and placenta followed the quality criteria described and the four parameters (F, F_total_, V_b_ and MTT) could be calculated (Fig 4).

In 40 cases (center 2), the kinetic curves of the AIF followed the quality criteria described. Nevertheless, the steady-state of the placental curves appeared above the steady-state of the AIF curves (S1B Fig). As this does not follow placental physiology, we did not include them in the analysis. In 5 other cases (center 3), the AIF curve did not decrease after the initial peak. DCE sequences revealed a lower than expected spatial resolution (pixel spacing 3.9 x3.9 mm) (see S1 File for details of pixel spacing). S1C Fig represents the corresponding kinetic curves of AIF and the placenta. These cases were excluded from our analysis as the data was degraded by partial volume averaging secondary to the low resolution.

### Dynamic sequences

The placenta, the amniotic fluid and the fetus were easily distinguished on FSPGR sequences. Enhancement of the placenta started from the zone in contact with the myometrium, and then propagated through the placenta. No enhancement of the fetus was visible on any of the exams, which is in line with our non-permeable one compartment model (Fig 3).

### Compartmental analysis and perfusion parameters

The one compartment analysis was performed for all 63 placentas (Fig 2).

One data-set was excluded as an outlier with very aberrant values of the parameters (greater than 4 standard deviations less than the mean). With the exclusion of this outlier, 62 total data sets were included in our analysis. Out of the 62 fetuses whose placentas were included in the study, 13 had IUGR without any chromosomal anomalies, 12 fetuses had chromosomal abnormalities without evidence of IUGR at birth, and 2 had both IUGR and chromosomal anomalies. Table 1 shows the characteristics and reasons for which terminations were requested and confirmed at post-mortem exam, in both groups. Pathological examinations of the placentas found significantly more lesions in the placentas of IUGR fetuses (n = 9; 69.2%) as compared to normally grown fetuses.

**Table 1:** Characteristics of the pregnancies. Three groups were compared: appropriate for gestational age birthweight fetuses AGA (n = 35); intrauterine growth restriction fetuses IUGR (n = 13) and abnormal karyotype fetuses (n = 14). Data were compared using t-test (*) to evaluate pathological abnormality or ANOVA for other values.

	AGA group n = 35	IUGR group n = 13	Abnormal karyotype group n = 14	P-value
GA at TOP (weeks)	26 (±4.9)	23 (±6.1)	24 (±2.9)	0.08
Birthweight (grams)	1083 (±696)	557 (± 535)	683 (±349)	<0.01
Birthweight (Z-score)	0.03 (± 0.89)	-2.48 (± 1.32)	-0.51 (±1.06)	<0.01
Placental weight (grams)	209 (±90)	149 (±103)	240 (± 31)	0.10
Significant placental pathological abnormality	6 (17.1)	9 (69.2)		<0.01 (*)

*Results are presented as mean* (SD) or effective (%).

#### *i*. Overall results

Among our study group (n = 62) mean perfusion parameters F, F_total_, V_b_ and MTT were 124±59 mL/min/100mL, 224±133 mL/min, 62±11% and 0.63±0.36 s respectively. These were 129±61 mL/min/100mL, 222±137 mL/min, 63±10% and 0.63±0.39 s and 109±50 mL/min/100mL, 246±96 mL/min, 62±13% and 0.65±0.36 s in the group with normal (n = 48) and abnormal (n = 14) karyotype, respectively.

#### *ii*. Perfusion parameters in the AGA and IUGR group with normal karyotype

Among AGA group (n = 35), mean perfusion parameters F, F_total_, V_b_ and MTT were 137±59 mL/min/100mL, 259±134 mL/min, 64±10% and 0.56±0.28 s respectively.

**Fig 5:**
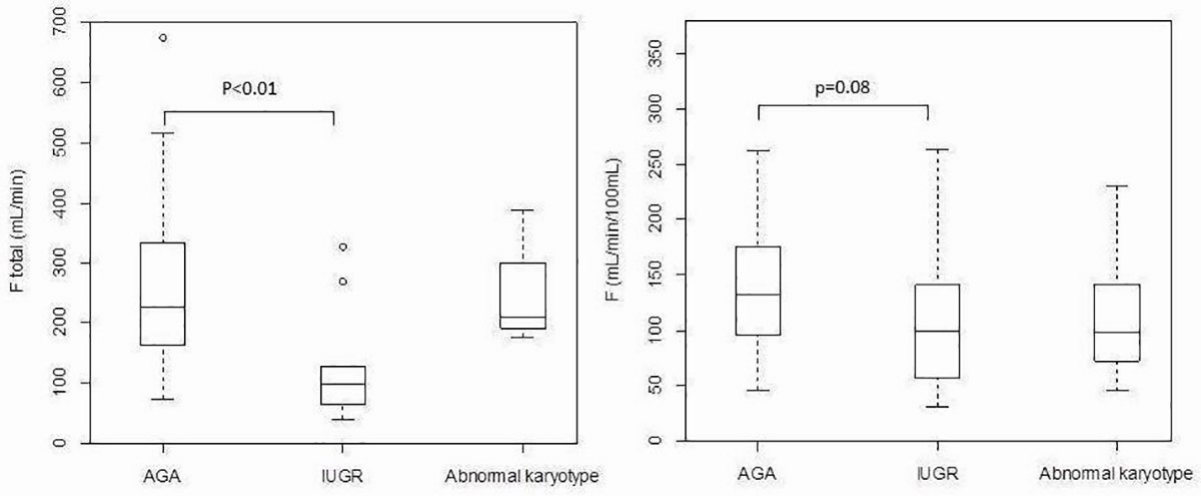
Box plot of placental perfusion F total (mL/min) and F (mL/min/100mL) as a function of fetus group (appropriate for gestational age birthweight fetuses AGA; intrauterine growth restriction fetuses IUGR or abnormal karyotype fetuses). The circles on the graph indicate values that fell outside the ranges included in the box plots.

Fetuses affected by IUGR (n = 13) showed significantly lower F_total_ values than AGA fetuses (n = 35) (F _total_ = 122±88 mL/min versus 259±134 mL/min, p = 0.002), they also tend to show lower F and Vb values (F = 106±63 ml/min/100mL versus 137±59 ml/min/100mL, p = 0.08; Vb 59±9% versus 64±10%, p = 0.13) and higher MTT (0.80±0.57 s versus 0.56±0.28 s, p = 0.06) (Fig 5).

#### *iii*. Relationships between perfusion parameters and GA

**Fig 6:**
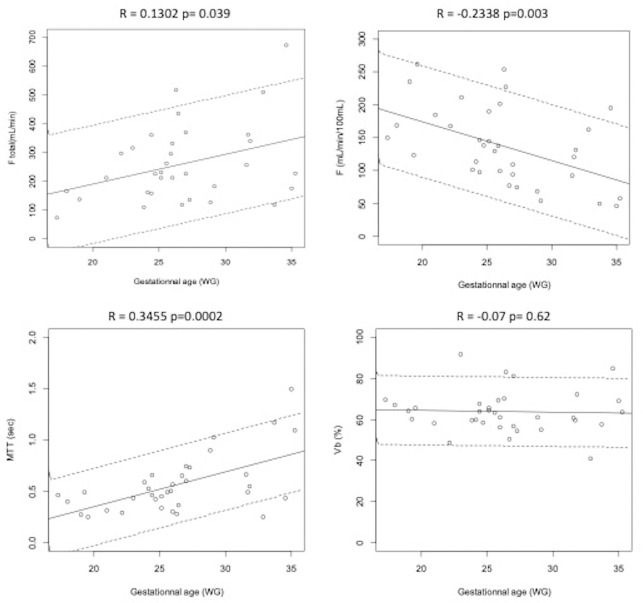
DCE MRI parameters as a function of the term of pregnancy in weeks gestational age (WG) among appropriate for gestational age fetuses AGA (n = 35). DCE perfusion parameters were presented: Ftotal: Total placental blood flow, in mL/min; F: Placental blood flow, in mL/min/100mL; MTT: Mean Transit Time, in seconds; Vb: Blood volume fraction of the maternal vascular placental compartment, in %. The solid line represents regression model and dotted line represents 5^th^ and 95^th^ confidence interval.

Among the study population of AGA (n = 35), perfusion parameters F, F_total_ and MTT showed a linear correlation with GA (r = -0.2338 p = 0.003; r = 0.13 p = 0.03; and r = 0.3455 p = 0.0002, respectively). No correlation was found between V_b_ and GA (r = -0.07 p = 0.62) (Fig 6).

## Discussion

Our preliminary study shows that DCE MRI of the placenta, a robust method to evaluate placental perfusion in animal models, could be technically feasible in pregnant women. Values of placental perfusion calculated using one-compartment modelling are in line with the physiology and growth of the placenta.

The Placentimage trial is the first study where in vivo functional imaging was evaluated in pregnant women using a Gd based contrast agent. To our knowledge, only a few studies have used Gd based contrast agent in pregnant women [18, 26–28]. In these earlier studies, MRI was used to describe the anatomy of the placenta in suspected cases of placental abnormalities. None of the authors described quantitative parameters of perfusion. Obtaining such functional imaging data in human pregnancy is no simple endeavor and is unlikely to be soon repeated.

In our study, F mean was 129±61 mL/min/100ml in cases with a normal karyotype. Interestingly, values of F were lower in IUGR pregnancies although this finding did not reach statistical significance. F total was significantly decreased in case of IUGR which is related to a significant decrease in placental weight in these fetuses. Given the fact that placental weight itself is correlated with birth weight [29], this strengthens the highly significant association between IUGR and F_total_ with a clear dose-effect. Thus, Ftotal shows that the placental weight differ between IUGR and AGA but does not reflect placental functional parameters, which should be explained by F. Two authors evaluated placental perfusion in humans and found very similar F mean values. In an earlier study, Bodis et al. [30] used isotope techniques to estimate placental perfusion and found F mean of 110 mL/min/100ml. Gowland et al. [31] performed echo planar imaging at 0.5 T in 15 patients and found F mean of 176±24 mL/min/100ml. Our results are also very similar to our previous results of placental perfusion determined by DCE MRI in mice (with placental perfusion ranging from 115 to 180 mL/min/100mL) [9–14]. F decreased with GA in pregnancy. Given that F was expressed in mL/min/100mL this trend can be explained. F at around 200mL/min/100mL at the beginning of the second trimester, is approximately halved by the end of the third trimester, this probably relates to villous maturation and decreased placental efficiency over gestation. Over the same period, the average placental weight increases approximately four-fold. It is therefore logical that the placental perfusion (F_total_) increases with GA, in line with growth of the placenta.

V_b_ mean was 63±10%. It is a greater value than that published in rodents (from 36% to 42%) [9–14]. It is also greater than the 35.3% obtained in a morphometric study of placenta sampled during caesarian section [32]. This could be a result of the morphometric studies not evaluating the microcirculation whereas our method looks at total blood volume including both micro and macro circulation volumes. V_b_ did not change either with the GA nor the fetal birth weight and appears as a stable parameter during pregnancy. MTT increased with the term of pregnancy. This parameter corresponds to the average time that the blood spends in a pre-determined volume and its evolution is in line with the increasing placental mass.

The Placentimage trial has several limitations and we must be careful in the interpretation of our results, which remain exploratory. Although we aimed at standardizing acquisition protocols among centers, there were some differences between centers and exams performed in centers 2 and 3 were excluded because they did not fulfill our quality criteria (S1B and S1C Fig). In center 2, difficulties were likely due to technical errors and perhaps absence of saturation bands. The absence of saturation bands affects the SNR, with gadolinium concentrations observed in the placenta that were higher than plasma concentrations. This made it impossible to calculate placental perfusion in these centers. The poor spatial resolution in center 3 likely resulted in poor performance on our quality assessment. This highlights that the DCE-based functional MRI is very sensitive to the specific and detailed MR imaging protocol and parameters in the experimental setting and that results from one center cannot simply be extrapolated to other centers: overall, less than half of recruited subjects actually had MRIs with interpretable data. Another important limitation, but unavoidable, is that we did not include any truly normal ongoing pregnancies resulting in live-births. As the safety of Gd-chelates has not been established in pregnancy, our trial only included pregnancies that were already scheduled for termination due to various fetal anomalies. However, despite this limitation, measured perfusion values in the cohort we defined as “normal” placentas is similar to other published values using non-contrast methods. Indeed, we have taken care to separately examine chromosomal abnormalities and to consider the absence of significant placental pathological lesions in the AGA group. While we have taken care to separately examine chromosomal abnormalities, and to analyse our results according to birth weight and the possible presence of placental pathological lesions, one can easily consider our population as a non-normal population as there was a wide spectrum of fetal anomalies (Table 1). Therefore, the placental perfusion parameters we report, even in cases of a pregnancy without IUGR, chromosomal, or placental abnormalities may not perfectly apply to a normally evolving pregnancy. This is even more relevant as placental disease has been reported to be more common in fetuses with abnormalities such as congenital heart disease [33]. However, these hypotheses remain to be substantiated. In addition, the values obtained in our study do show large variation. It is unclear whether these variations reflect biological variances, which could reflect the various pathologies of the fetuses, or if they reflect lack of precision inherent to our technique. While uterine artery ligations and noradrenaline injection are some methods used in animal studies to mimic decreased placental perfusion, the actual pathology associated with placental insufficiency in humans is likely very different and occurs more at the utero-placental interface and spiral arteries than as a reduction in overall flow. Furthermore, placental pathologies can be focal or diffuse. Finally, an obvious limitation of this trial was the use of a Gd based contrast agents. The use of this contrast agent is not recommended routinely during pregnancy [34]. It is used in very few and specific situations when a placental insertion abnormality is suspected [18]. However, while the use is not established as safe, there is also very little literature on the teratogenicity and pharmacokinetics of Gd in human pregnancies [35]. Gd assay analysis, performed during the Placentimage trial, should provide more insights into maternal-fetal leakage of Gd and these results will be published in the near future. Deloison et al. [13] showed that placental perfusion could be measured in vivo by using iron oxide nanoparticle based contrast agents (SPIO, superparamagnetic iron oxide) in rats. Liposome-based Gd agents which do not cross the placenta [36] or SPIO based agents may be more suitable for use in pregnant women but their safety has also not yet been established in human pregnancies. Arterial spin labeling (ASL) and/or intravoxel incoherent motion (IVIM) have also been used to measure placental perfusion in pregnant women [37–39]. ASL or IVIM could overcome the limitation of contrast agents as these imaging techniques do not require the use of an exogenous contrast agent.

Although use of Gd based contrast agents in pregnant women is currently very limited [34], DCE-based techniques remain a robust method to measure in vivo perfusion of organs and our study paves the way and provides a benchmark for future developments in placental perfusion studies.

## Conclusion

Our exploratory study shows that measuring placental perfusion in vivo is possible by DCE MRI and suggests that total placental perfusion is altered in IUGR pregnancies consistent with decreased placental volume. There is a trend for impairment of other placental perfusion parameters that placental perfusion is altered placental functional parameters are altered in IUGR pregnancies. This study gives the first DCE MRI values that provide a potential standard for future research into placental perfusion methods. However, it has a number of acknowledge limitations, such as technical, study population, and limited reproducibility between centers.

## Supporting information

S1 Fig Kinetic curves observed in the center 1 (a), in the center 2 (b) and in the center 3 (c).
(TIFF)

S1 File (DOCX)
