# Supplementary figures and images for: Human placental perfusion measured using dynamic contrast enhancement MRI
Source: PLoS One. 2021 Sep 2;16(9):e0256769. doi: 10.1371/journal.pone.0256769 (PMC8412340; doi:10.1371/journal.pone.0256769)

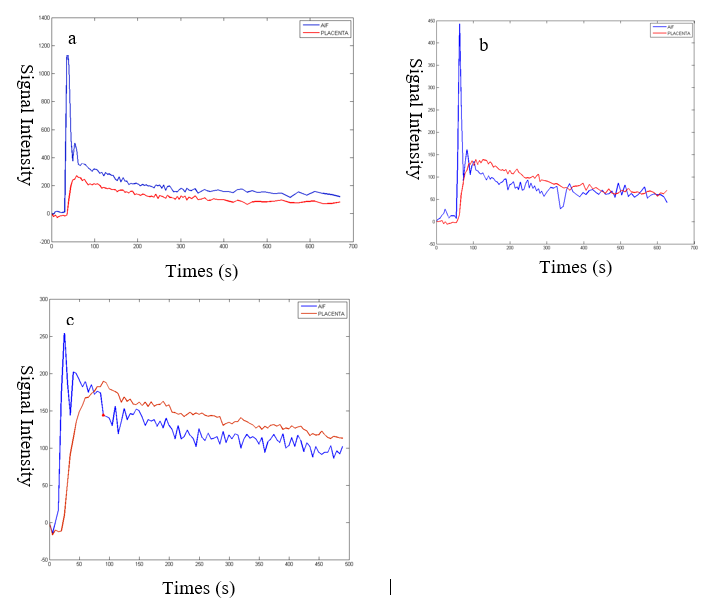

Supplement: S1 Fig — Kinetic curves observed in the center 1 (a), in the center 2 (b) and in the center 3 (c). (TIFF) [file pone.0256769.s001.tiff]
