# Supplementary material for: Human placental perfusion measured using dynamic contrast enhancement MRI
Source: PLoS One. 2021 Sep 2;16(9):e0256769. doi: 10.1371/journal.pone.0256769 (PMC8412340; doi:10.1371/journal.pone.0256769)
Supplement: S1 File — (DOCX) [file pone.0256769.s002.docx]

# S1 File

***Segmentation method***

Signal Intensities (SIs) were measured by regions of interests (ROIs) over the aorta (or iliac artery, when the aorta was not visible on the selected axial slice) as well as for the entire placenta.

ROIs were placed on the first slice of the placenta and automatically extended across all slices to capture the entire placental volume. Manual adjustments were made to exclude non-placental regions captured by the software’s automatic propagation and ROIs were automatically propagated across the time axis. This was performed using a cluster method based on K means.

ROIs were placed over the aorta (or iliac artery) and automatically propagated across the time axis. To prevent a partial volume averaging effect, pixels at the edge of the ROI were excluded and only the pixels with the highest enhancement peak were selected.

For each studied structure, SIs were represented as a function of time. Two kinetic curves were obtained for analysis: the arterial kinetic curve (AIF) and the placental kinetic curve (Supplemental Figure 1).

***Detailed Parameters of the DCE MRI sequences in the different centers***

*Center 1*

MRI studies were performed on a Signa, HDxt, GE medical system, Buc, France.

FIESTA sequence parameters were as follows: Repetition Time (TR): 3.46 ms, Echo Time (TE): 1.12 ms, Flip Angle (FA): 50°, matrix 320 x 256, slice thickness 5 mm, inter- slice gap 5 mm.

DCE sequence parameters were as follows: TR: 1.996 ms TE: 0.824 ms, FA 15°, matrix 128x128 *(**reconstruction 256x256)*, FOV: 82x82 cm ; slice thickness 5 mm, inter-slice gap 5 mm, voxel size 1.875x1.875x 5mm. 20 slices were acquired over 99 phases (1980 images). Temporal resolution: 4 to 5 seconds in the first eighty phases, and 10 to 15 seconds for the final phases.

*Center 2*

MRI studies were performed on a Signa, MR Discovery 450, GE medical system, Buc, France.

FIESTA sequence parameters were as follows: TR: 3.65 ms, TE: 1.6 ms, FA: 65°, matrix 224 x 224, slice thickness 6 mm, spacing between slice 7 mm.

DCE sequence parameters were as follows: TR 2.168 ms and TE 0.884 ms, FA 15°, matrix 128x128 *(reconstruction= 512x512)*, FOV 40x40 cm, slice thickness 5 mm, inter-slice gap 2.5 mm, voxel size 0.9mmx0.9mmx5mm. 64 slices were acquired during 70 or 99 phases (4480 or 6336 images). Temporal resolution: from 4 to 5 seconds at the beginning of the exam, and extending over 10 to 15 seconds at the conclusion.

*Center 3*

MRI studies were performed on an Avanto 1.5T system, Siemens medical systems Germany.
True-FISP sequence parameters were as follows: TR: 4.15 ms, TE: 1.67 ms, FA: 60°, matrix 448 x 321, slice thickness 8 mm, spacing between slices 8 mm.

DCE sequence parameters were as follows: TR 12.4 ms and TE 4.78 ms; FA 15°; matrix 128x66 *(**reconstruction 128x96)*, FOV 43x32 cm slice thickness 5 mm, voxel size 3.9mmx3.9mmx5 mm. 20 slices were acquired during 99 phases (1980 images). Temporal resolution: from 4 to 5 seconds in the first eighty phases, and 10 to 15 seconds in the final 19.

*Center 4*

MRI studies were performed on an Intera 1.5T, Philips medical system, Netherlands.

Balanced-FFE sequence parameters were not available.

DCE sequence parameters were as follows: TR 15.85 ms and TE 3.398 ms, FA 70°, matrix 128x115 *(reconstruction 256x256*), FOV 40x40 cm, slice thickness 5mm, spacing between slices 15 mm, voxel size 1.8mmx1.8mmx5 mm. 5 slices were acquired over 150 phases (750 images). Temporal resolution was 4 seconds during the entire acquisition.
